# Supplementary material for: Resolving the full spectrum of human genome variation using Linked-Reads
Source: Genome Res. 2019 Apr;29(4):635–45. doi: 10.1101/gr.234443.118 (PMC6442396; doi:10.1101/gr.234443.118)
Supplement: Supplemental Material [file supp_gr.234443.118_Supplemental_longranger-2.2.zip › longranger-2.2.tar.bz2!/lr2.2-public/lib/stan/cmdstan/stan/doc/api/html/base__adapter_8hpp_source.html]

Stan: src/stan/mcmc/base\_adapter.hpp Source File


|  |  |
| --- | --- |
|  | Stan  2.5.0  probability, sampling & optimization |


- Main Page
- Related Pages
- Namespaces
- Classes
- Files

- File List
- File Members

All Classes Namespaces Files Functions Variables Typedefs Enumerations Enumerator Friends Macros Pages

- src
- stan
- mcmc

base\_adapter.hpp

Go to the documentation of this file.

1 #ifndef STAN\_\_MCMC\_\_BASE\_\_ADAPTER\_\_BETA

2 #define STAN\_\_MCMC\_\_BASE\_\_ADAPTER\_\_BETA

3

4 namespace stan {

5

6  namespace mcmc {

7

8  class base\_adapter {

9

10  public:

11

12  base\_adapter(): adapt\_flag\_(false) {};

13

14  virtual void engage\_adaptation() { adapt\_flag\_ = true; }

15  virtual void disengage\_adaptation() { adapt\_flag\_ = false; }

16

17  bool adapting() { return adapt\_flag\_; }

18

19  protected:

20

21  bool adapt\_flag\_;

22

23  };

24

25  } // mcmc

26

27 } // stan

28

29 #endif

stan::mcmc::base\_adapter::engage\_adaptation

virtual void engage\_adaptation()

**Definition:** base\_adapter.hpp:14

stan::mcmc::base\_adapter::base\_adapter

base\_adapter()

**Definition:** base\_adapter.hpp:12

stan::mcmc::base\_adapter::adapt\_flag\_

bool adapt\_flag\_

**Definition:** base\_adapter.hpp:21

stan::mcmc::base\_adapter

**Definition:** base\_adapter.hpp:8

stan::mcmc::base\_adapter::adapting

bool adapting()

**Definition:** base\_adapter.hpp:17

stan::mcmc::base\_adapter::disengage\_adaptation

virtual void disengage\_adaptation()

**Definition:** base\_adapter.hpp:15


---

[ Stan Home Page ]

*© 2011–2014,
Stan Development Team.*
